# Encoding models for developmental cognitive computational neuroscience: Promise, challenges, and potential

**DOI:** 10.1016/j.dcn.2024.101470

**Published:** 2024-10-30

**Authors:** Tomoya Nakai, Charlotte Constant-Varlet, Jérôme Prado

**Affiliations:** a Centre de Recherche en Neurosciences de Lyon (CRNL), INSERM U1028 - CNRS UMR5292, Université de Lyon, France; b Araya Inc., Tokyo, Japan

**Keywords:** Cognitive computational neuroscience, Encoding model, Artificial neural network, Development, FMRI, EEG

## Abstract

Cognitive computational neuroscience has received broad attention in recent years as an emerging area integrating cognitive science, neuroscience, and artificial intelligence. At the heart of this field, approaches using encoding models allow for explaining brain activity from latent and high-dimensional features, including artificial neural networks. With the notable exception of temporal response function models that are applied to electroencephalography, most prior studies have focused on adult subjects, making it difficult to capture how brain representations change with learning and development. Here, we argue that future developmental cognitive neuroscience studies would benefit from approaches relying on encoding models. We provide an overview of encoding models used in adult functional magnetic resonance imaging research. This research has notably used data with a small number of subjects, but with a large number of samples per subject. Studies using encoding models also generally require task-based neuroimaging data. Though these represent challenges for developmental studies, we argue that these challenges may be overcome by using functional alignment techniques and naturalistic paradigms. These methods would facilitate encoding model analysis in developmental neuroimaging research, which may lead to important theoretical advances.

## 1 Introduction

There is a growing interest in understanding the computational principles underlying perception, action, and cognition (Kriegeskorte and Douglas, 2018, Naselaris et al., 2018). This emerging field of research, sometimes called, “cognitive computational neuroscience (CCN),” aims to bridge the gap between cognitive science, neuroscience, and artificial intelligence. In doing so, it may provide a framework for interdisciplinary research, such that cognitive theories and neural data may impose biologically plausible constraints on artificial intelligence systems while artificial intelligence may make testable predictions regarding neuro-cognitive theories.

Despite that growing interest, CCN has seldom been applied to the study of neuro-cognitive development, with the exception of a few disorder classification studies that have made use of state-of-the-art artificial neural networks (ANNs) (Joshi et al., 2023, Tomaz Da Silva et al., 2021). Yet, by using non-invasive neuroimaging techniques such as functional magnetic resonance imaging (fMRI), magnetoencephalography (MEG), electroencephalography (EEG), and near-infrared spectroscopy (NIRS), cognitive neuroscientists have collected over the years a large amount of data. We argue that a computational approach is in a unique position to make use of such data to investigate how neuro-cognitive mechanisms may change dynamically with learning and development (Gilmore et al., 2018, Peelen and Downing, 2023, Thomas et al., 2019). For instance, Peelen and Downing (2023) have argued that cross-format decoding may inform cognitive theories as it allows researchers to test whether brain representations are shared between different stimulus types (e.g., symbolic and nonsymbolic representations of numbers; Nakai et al., 2023) and whether such a representational relation changes with exposure to formal education (Nakai et al., 2023).

Critically, artificial intelligence research has shown that ANNs trained on large-scale datasets may exhibit levels of cognitive ability that appear relatively similar to those exhibited by humans (Fei et al., 2022, Korteling et al., 2021). There have been concerns that ANNs require much larger amounts of input than human infants (Frank, 2023). Yet, recent studies have shown that an ANN trained with a small dataset of visual and language inputs from one child (Davidson et al., 2024, Vong et al., 2024) can successfully learn the word-object associations and spatial relations of objects (such as “above” and “below”) that children learn in everyday life. Such studies indicate that ANNs are potentially useful models simulating the development of human cognitive abilities. However, a fundamental question is whether ANNs and human brains exhibit computational processes and developmental trajectories that are comparable. Therefore, there is little doubt that developmental neuroimaging research can contribute to progress in CCN, for at least two reasons. First, it may enable quantitative modeling of dynamic changes in brain activity patterns that underlie cognitive development. Second, it can provide valuable insights for building artificial intelligence based on mechanisms similar to those of human development and learning.

The goal of this review is to examine how CCN may be applied to developmental neuroimaging. We first outline so-called *encoding models*, one of the main methods of CCN, and describe how they can contribute to our understanding of neuro-cognitive development. We then present some of the recent attempts that have combined encoding models with developmental neuroimaging data. We point out the caveats and difficulties in applying encoding models to developmental data, and provide an overview of the technical advances that could contribute to the future development of this field.

## 2 Encoding versus decoding models

**Fig. 1:**
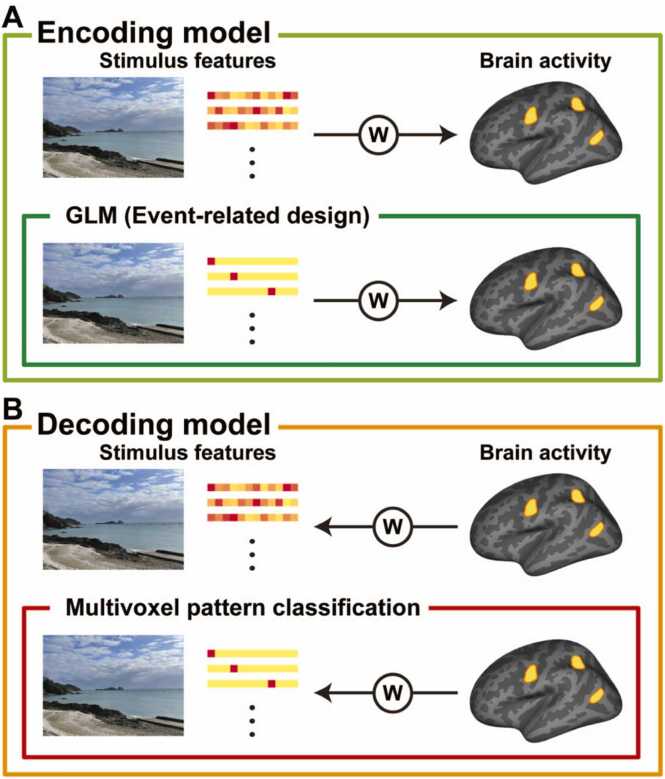
Difference between encoding and decoding models. (A) Encoding models, including the general linear model (GLM) under the event-related design, use stimulus features as input and brain activity as output. (B) Decoding models, including multivariate pattern classification, use brain activity as input and stimulus features as output. W indicates model weights.

Encoding models are an important method within CCN (see Kriegeskorte and Douglas 2018 for the other approaches)*.* In an encoding model analysis, brain activity is predicted by a weighted linear combination of features extracted from experimental stimuli (Fig. 1**A**). In other words, encoding models use stimulus features as input and brain responses as output. It is interesting to consider that the general linear model (GLM), adopted in the statistical parametric mapping approach (Friston et al., 1994), is a special case of an encoding model where stimulus features consist of categorical labels of some experimental conditions (i.e., event-related design). Although earlier studies have used hand-crafted features to build encoding models (Huth et al., 2012, Kay et al., 2008, Nishimoto et al., 2011), recent studies have developed encoding models based on latent features derived from various ANN models (Goldstein et al., 2022, Güçlü and van Gerven, 2015, Khosla et al., 2021, Nakai and Nishimoto, 2023a). Encoding models thus serve to link brain activity and artificial intelligence through a form of representational space and enable testing cognitive theories on this space (Kriegeskorte and Douglas, 2018).

**Fig. 2:**
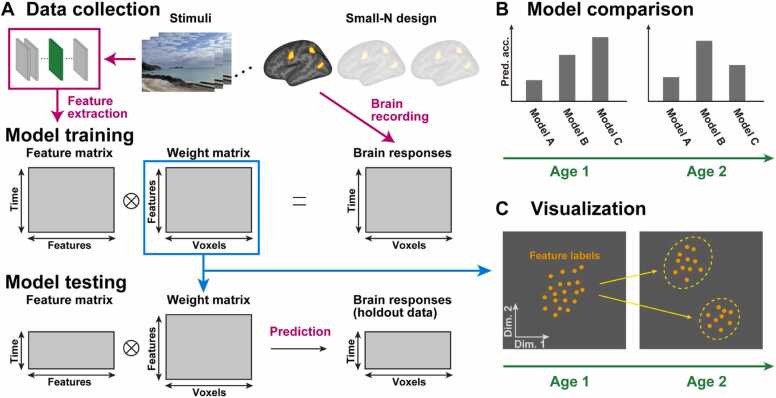
Pipeline of encoding model analysis. (A) A feature matrix is obtained by extracting features from experimental stimuli. Naturalistic stimuli (such as movies) and artificial neural networks can be used in this step. A brain response matrix is prepared for each subject (under the small-N design). An encoding model is (in most cases) trained using regularized linear regression between the feature and brain response matrices. The resultant weight matrix is further used in the model testing and visualization steps. Model performance is evaluated by predicting brain responses of holdout test data. (**B**) Prediction accuracy can be compared across different models (Model A to C) and different ages (e.g., Age 1 and Age 2). Note that brain activity may be measured at different ages within the same individual (i.e., in longitudinal designs) or between individuals (i.e., in cross-sectional designs). (**C**) Brain representations of target features (indicated by orange dots) can be visualized on two-dimensional space for different age groups. Two feature clusters that appear at Age 2 are circled in yellow.

In practice, encoding models often predict brain activity at each cortical voxel (i.e., voxel-wise encoding models) or surface vertex (i.e., vertex-wise encoding models) from a linear combination of multivariate features extracted from stimuli multiplied by weight coefficients (Fig. 2**A**). Experimental tasks are often passive presentations of naturalistic stimuli such as visual images (Güçlü and van Gerven, 2015, Kay et al., 2008, St-Yves et al., 2023), movies (Khosla et al., 2021, Koide-Majima et al., 2020, Nishimoto et al., 2011), speech (Goldstein et al., 2022, Huth et al., 2016, Nakai et al., 2021b), and music pieces (De Angelis et al., 2018, Nakai et al., 2021a). To avoid overfitting to training data, researchers often use some regularized regression algorithms to estimate model weights (e.g., ridge regression; Hoerl and Kennard, 1970). Features extracted from the holdout test data stimuli are multiplied by the model weights obtained during training to predict brain activity of the test data, and model performance (prediction accuracy) is quantified using correlation coefficients or coefficients of determination between predicted and actual brain activities (Schoppe et al., 2016). Nonparametric permutation testing is generally advised when testing for statistical significance in a cross-validation approach, because the results from each cross-validation fold are not independent and the degrees of freedom for parametric statistics are overestimated (Scheinost et al., 2019).

Encoding models are interpreted based on the concept of *linearizing feature space* (Naselaris et al., 2011), which assumes that the relation between brain activity and features is linear even though the relation between stimulus input and features can be nonlinear. This indicates that the interpretation of results from an encoding model is highly dependent on how the features are preprocessed. However, encoding models benefit from such preprocessing biases because feature preprocessing reflects researcher’s hypothesis about brain representations. Typically, researchers do not test every feature available. They selectively choose a few features to test a hypothesis about brain representations. For example, Nakai and Nishimoto (2023a) showed that large language ANN features predicted brain activity of math problems better than semantic and visual features. This is consistent with the hypothesis that human subjects would solve math problems using language functions that rely on long-distance dependency (Matsumoto and Nakai, 2023).

To some extent, encoding models are defined in opposition to *decoding models*, which use brain responses as input and stimulus features as output (Fig. 1**B)** (Kriegeskorte and Douglas, 2019, Naselaris et al., 2011). Since decoding models link features and responses in the opposite direction as encoding models, weight coefficients are assigned to brain activity instead of stimulus features. Multivoxel pattern classification, for example, can be regarded as a subset of decoding models where the input data consists of activity patterns of multiple voxels but the output is discrete labels (such as stimulus categories or task conditions). In other words, decoding models can take continuous latent features as the output in its general form. For example, by returning latent representations of a decoding model to the ANN, it can generate visual images from brain activity (Takagi and Nishimoto, 2023). Decoding models are a promising approach in terms of real-world applications such as brain-machine interfaces, but they are not optimal for investigating which brain regions represent target cognitive functions (with perhaps the exception of searchlight analysis; Kriegeskorte et al., 2006) because the output of decoding models is stimulus features rather than brain activity. Therefore, the application of CCN to developmental neuroimaging is more likely to involve encoding models, which are the focus of the present review.

## 3 Why are encoding models important in developmental research?

The use of encoding models can provide numerous benefits to developmental cognitive neuroscience. First, encoding models could provide a quantitative evaluation of cognitive components contributing to the brain activity of different subject populations and age groups (Fig. 2**B**). Prediction performance of different features can be compared by applying the encoding models to the test data not used during training. Features that exhibit better prediction performance are likely to be more similar to the information represented by the brain. For example, Nakai et al. (2021a) compared prediction accuracy of five different acoustic models and showed that a biologically plausible spectro-temporal modulation transfer function model was most accurate in predicting brain activity during a music listening task. de Heer et al. (2017) compared the prediction performance of spectral, phonetic, and semantic encoding models and found their distinct contributions from the primary auditory area to the lateral temporal cortex. In the case of constructing encoding models for multiple subjects, group-level difference of prediction accuracies can be assessed using statistical tests. For example, Jessen et al. (2019) used *t*-tests to compare prediction accuracies of auditory envelope and visual motion features for movie-viewing EEG data. Applying this methodology to developmental data would allow for the comparison of prediction accuracy at different age groups and for the investigation of dynamic changes in brain representations of multiple cognitive functions during development.

Second, encoding models are suitable for analyzing developmental neuroimaging data in naturalistic paradigms. Naturalistic paradigms, such as movies, speech, and music, have gained increasing attention in recent years as stimuli with more ecological validity compared to conventional experimental designs (Jääskeläinen et al., 2021, Tervaniemi, 2023). Recent studies have reported better accuracy in estimating individual differences with movie viewing fMRI data compared to resting-state fMRI data (Finn and Bandettini, 2021, Gal et al., 2022). Naturalistic paradigms do not impose complex instructions and can reduce head motion compared to resting state (Greene et al., 2018, Vanderwal et al., 2019), making it more suitable for experimentation with young children. One challenge in analyzing naturalistic stimuli, however, is their complexity. Indeed, they cannot be decomposed and modeled using discrete categorical features as in the event-related design. Encoding models are effective for analyzing naturalistic stimuli based on latent features. For example, Nishimoto et al., (2011) constructed encoding models using latent visual features extracted through various spatiotemporal filters to predict brain activity of three subjects watching movie stimuli. In Huth et al., (2016), brain activity of seven subjects listening to story stimuli was modeled using latent semantic features based on word co-occurrence statistics. Since a variety of features can be extracted from the same stimuli, encoding models can expand the possibilities for analyzing developmental data under the naturalistic paradigm.

Third, encoding models can incorporate latent features from ANNs and allow for comparisons in the development of artificial and biological neural networks. ANN consists of multiple layers of neuronal units, where the weighted sum of units in one layer is used as input for the next layer after a nonlinear transformation. By using experimental stimuli as input and extracting the output of the intermediate layers as vector embeddings, these vectors can be used as latent features of encoding models. For example, Güçlü and van Gerven (2015) showed that encoding models based on latent features of ANNs (trained with visual object categorization) predicted brain responses in the human visual cortex. Such correspondence was hierarchically organized; i.e., the early visual cortex corresponded to the shallower ANN layers. Assuming a linearization of feature space (Naselaris et al., 2011), this suggests that nonlinear transformations accumulated from the input stimuli to deeper layers led to a linear relation with brain activity at a certain layer. The results of Güçlü and van Gerven (2015) are thus consistent with the hierarchical organization of the human visual system (Grill-Spector and Malach, 2004) and may bridge the gap between ANNs and the human brain. Similar analyses can be performed on neuroimaging data of children at different stages of development. Recent studies constructed ANNs based on visual and verbal input similar to that of one child aged 6–25 months (Davidson et al., 2024, Vong et al., 2024). Although these studies have primarily examined the output of ANNs, encoding models could extend their concepts to include comparisons between internal representation of ANNs and the developing brain. Encoding models could thus provide an interpretation of ANNs, which are sometimes called “black box”, in terms of their similarity to biological neural networks.

Fourth, weight coefficients of encoding models could be further analyzed to visualize the developmental trajectory of brain representations (Fig. 2**C**). Dimensionality reduction techniques such as principal component analysis enable mapping brain representations, which sometimes have hundreds of dimensions, onto two- or three-dimensional space and facilitate intuitive interpretation. For example, Huth et al. (2016) used principal component analysis to the weight matrix obtained using fMRI data of a story listening task, and visualized how diverse semantic concepts are organized on the 2D maps generated by different principal components in each cortical voxel. Koide-Majima et al. (2020) visualized how representations of 25 emotion dimensions during movie viewing are modulated on the cortical surface. Even if encoding models may accurately predict developmental data, a common critique is that their internal structure may appear to be a black box. However, visualization techniques help interpret how brain representations dynamically change with development. Brain representations that are similar to each other at a certain age may separate into two clusters at another age (Fig. 2**C**). We argue that such data-driven visualization could lead to new discoveries about developmental changes in brain representations and could help bridge the gap between prediction and explanation (Shmueli, 2010).

The above characteristics of the encoding model are likely to open up new research opportunities in developmental cognitive neuroscience. For example, encoding models make it possible to compare prediction accuracy of ANN-based encoding models across different age groups, or track these changes within individuals over time, testing whether neural processing becomes more similar to ANN models with development. Visualization coming from encoding models may also be useful to determine how different stimuli are represented across development. For example, it might be used to examine how different phonemes of native and non-native languages are represented across early childhood (Menn et al., 2023) or whether different types of arithmetic operations are represented differently across development (Istomina and Arsalidou, 2024). Encoding models may also be useful to investigate neurodevelopmental disorders. For instance, by applying encoding models to brain activity of dyslexic children while listening to natural speech, it may be possible to determine which of the phonological, syntactic, and semantic features have reduced prediction performance or altered representations compared to typically developing children. In sum, encoding models may allow researchers to go largely beyond conventional analyses (such as GLM), which may provide new insights into developmental research.

## 4 Previous encoding model research with developmental data

Not many studies have attempted to use encoding models in analyzing developmental neuroimaging data. By far, these models have been most frequently used with EEG and MEG data. In M/EEG analysis, encoding models are often called (multivariate) *temporal response function* (TRF) models (Brodbeck et al., 2023, Crosse et al., 2016). TRF models predict brain activity by linear combinations of stimulus features multiplied by weight coefficients. Weight coefficients (i.e., TRFs) are often estimated using regularized linear regression with training samples (but also see Brodbeck et al. 2023 for other possible approaches), and model performance is evaluated in holdout test samples. Using this approach to seven-month-old infants, Kalashnikova et al. (2018) trained a TRF model based on the speech envelope extracted from stimuli used during EEG experiments and showed different model weights between infant-directed speech and adult-directed speech in the left frontal areas. Jessen et al. (2019) constructed TRF models using EEG data from 7-month-old children watching a 5-min movie, combined with auditory, motion, and luminance features. By comparing models trained for each subject and those trained across subjects, they showed that TRF model prediction can be generalized to other individuals. Di Liberto et al. (2023) further built TRF models with a longitudinal cohort of infants at 4, 7, and 11 months of age. TRF models based on phonetic features showed an increase in the prediction accuracy of EEG signals during the first year of life, showing the effectiveness of this method in analyzing how pre-verbal infants learn phonetic information. Using cross-sectional EEG data from children aged 3 months to 4.5 years, Menn et al. (2023) found that prediction performance by phonetic features increased with age, specifically when children were listening to speech in their native language. All of these TRF model studies adopted naturalistic stimuli combined with continuous features, which cannot be used in the conventional event-related design. Moreover, Di Liberto et al. (2023), as well as their previous approach using the same dataset (Attaheri et al., 2022), is a unique example of how encoding models can contribute to revealing developmental dynamics of brain representations using longitudinal data, demonstrating the potential utility of encoding model approach in developmental cognitive neuroimaging.

Compared to studies using M/EEG, much fewer studies have applied encoding models to developmental neuroimaging datasets. This is nevertheless the case of two studies. First, Kamps et al. (2022) predicted movie viewing fMRI data of children aged 3–12 years in the fusiform face area and parahippocampal place area using encoding models trained with ANN features developed in their previous study (Ratan Murty et al., 2021). Second, Im et al. (2024) trained voxel-wise encoding models using the same fMRI dataset but with visual and social-affective features. They found that, while visual features predicted similarly across different age groups, the prediction accuracy of social-affective features increased with age. These two studies provide examples of the contribution of encoding models to developmental neuroimaging in terms of comparing ANNs and brain representations and different developmental stages based on prediction accuracy.

Despite the equivalence between the TRF model applied to M/EEG data and the encoding model that may be applied to fMRI data, there are some analytical differences between these models. In fMRI encoding models, weight values obtained during model training are often further analyzed using visualization methods such as principal component analysis (Huth et al., 2016, Koide-Majima et al., 2020). This allows for examining how representations of features or task categories are modulated across the cortex. Such detailed visualization attempts are rarely performed in TRF modeling studies, though they can be done. For example, Di Liberto et al. (2021) used a visualization analysis based on the TRF model. Using EEG data from native Mandarian speakers with varied English proficiency (L2) and native English speakers (L1), this study mapped regression weights of phonetic features from both subject groups on the two-dimensional space obtained using multidimensional scaling. With increased proficiency, the representational distance of phonemes between L1 and L2 subjects became shorter. Another marked difference between encoding models and TRF models is the target cognitive domains. The majority of studies using M/EEG-based TRF models have focused on auditory or phonological stimuli (Di Liberto et al., 2023, Liberto et al., 2021, Jessen et al., 2019, Kalashnikova et al., 2018). In contrast, fMRI-based encoding and decoding models have been mainly developed for visual processing (Huth et al., 2012, Kay et al., 2008, Naselaris et al., 2009, Nishimoto et al., 2011) and later applied to auditory and language processing (de Heer et al., 2017, Huth et al., 2016, Kell et al., 2018, Nakai et al., 2021b, Tang et al., 2023). In particular, it is worth highlighting that encoding models are increasingly being used to analyze complex cognitive functions such as mathematics (Nakai and Nishimoto, 2023a, Nakai and Nishimoto, 2023b). Application of TRF models to other cognitive functions would help to broaden the scope of developmental CCN research using M/EEG.

## 5 Challenges in applying encoding model analysis in developmental fMRI data

Many fMRI studies using encoding models (predominantly with adult subjects) are based on data collected with a small number of subjects but with a large number of samples per subject (small-N design) (Smith and Little, 2018) (Fig. 2). For example, Nakai and Nishimoto (2020) trained encoding models from six adult subjects who underwent three hours fMRI experiments (within-subject sample size/number of scans = 3748). A recent study has trained encoding models using 7 T fMRI data of eight adult subjects viewing 9000–10,000 natural images (three times each image; 22,000–30,000 trials in total; 30–40 h for each subject) (Allen et al., 2022, St-Yves et al., 2023). This sampling approach is taken because model prediction accuracy generally depends on the number of training samples. By comparing encoding models with different within-subject sample sizes, Antonello et al. (2023) revealed that prediction accuracy scales logarithmically with within-subject sample size. Matsuyama et al. (2023) replicated this finding in the visual domain. Wen et al. (2018) reported that such an increasing trend does not stop even at 10 h of training samples, suggesting that at least 10 h fMRI experiment may be needed for each subject. These studies suggest the importance of within-subject sample size in constructing encoding models.

Such a focus on within-subject sample size contrasts with conventional neuroimaging studies that have rather focused on across-subject sample size (i.e., the number of subjects), and relatively few studies have considered the impact of within-subject sample size (Chen et al., 2022, Nee, 2019). Ever since the emergence of noninvasive neuroimaging techniques, the standards for across-subject sample size have become progressively more stringent (Szucs and Ioannidis, 2020). Further, a recent study has demonstrated that measuring reliable brain-behavior association would require thousands of subjects (Marek et al., 2022), casting doubt on the majority of neuroimaging studies based on smaller across-subject sample size. On the other hand, researchers in CCN have discussed the advantages of collecting massive neuroimaging data for each individual (Hasson et al., 2020, Naselaris et al., 2021). Building comprehensive models of brain responses in natural environments requires broad sampling that fills the high-dimensional feature space as much as possible. Adding more subjects may increase noise, which hinders the goal of building such a model.

It should be noted, however, that individual variability of brain representations still contains rich information. This variability can be taken into account in encoding models. While several studies have attempted to enhance across-subject generalization in encoding model analysis (as discussed in the next section) (Güçlü and van Gerven, 2017, Wen et al., 2018), it is possible to quantitatively evaluate individual variability in brain representational space by comparing model weights across multiple subjects (Nakai et al., 2024). Such an analysis can reveal, for example, that brain representations of visual and language tasks are close for some subjects, while other subjects have close representations of auditory and language tasks. This approach, when combined with developmental data, may further reveal differences in how brain representations change from person to person. However, considering the limited experimental cost, there is a trade-off between within-subject sample size and between-subject sample size (Naselaris et al., 2021). Extensive sampling of a small number of subjects can be viewed as a preliminary step to future attempts to make inferences about individual differences.

Despite the growing interest in within-subject sample size, conducting long testing with children is challenging in neuroimaging research. Children often drop out due to fatigue and boredom. In the case of MRI, fear of noise and confined MRI equipment is also a major drop-out factor. Finally, developmental fMRI data often suffer from a greater amount of head motion than adult data, which increases the risk of data loss. Not surprisingly, the effect of head motion depends on the subject’s age. For example, younger children (5–10 years) display larger head motion than older children (11–15 years) during a movie-watching task (Greene et al., 2018). The risk of such withdrawal and data loss increases with the experimental duration, resulting in a smaller within-subject sample size compared to adult subjects. For example, in Nakai et al. (2023) testing task-fMRI with 5-year-old children, the within-subject sample size was n = 156. In the public dataset of movie-viewing fMRI experiment with 3–12 year-old children in Richardson et al. (2018), the within-subject sample size was n = 168. The two previous fMRI encoding models with developmental data (Im et al., 2024, Kamps et al., 2022) are based on this dataset. Compared to the sample size required for constructing encoding models for adult subjects, those with children are an order of magnitude smaller.

Another possible obstacle for developmental encoding models is the relative lack of task-based neuroimaging data compared to the resting state data. Encoding models are generally built upon task-based neuroimaging data because they aim to predict brain activity from features extracted from experimental stimuli. This analysis cannot be performed on structural MRI or resting-state fMRI data, both of which are widely used in neuroimaging studies with young children (Vijayakumar et al., 2018, Zhang et al., 2019). Clearly, acquiring task-based data is possible in children, but it is more challenging in children than in adults. This is because of a number of factors, including difficulty in providing complex instructions to children, fatigability, or the longer time required to respond to trials than adults (resulting in fewer available trials) (Kail, 1991).

Note that the above argument is mainly concerned with fMRI-based encoding models and does not apply to the EEG-based TRF models. TRF model studies do not fall under the small-subject design and generally perform statistical testing in a cross-subject manner. For example, Jessen et al. (2019) compared children and adults’ response functions at each sampling point using *t*-test. Di Liberto et al. (2023) tested group-level differences in prediction accuracy across different ages using repeated measures analysis of variance. Conversely, it remains unclear how accurately EEG data can be modeled for each individual. Such questions may be answered by using a recent massive individual EEG dataset (Gifford et al., 2022).

## 6 Possible solution and future implications

In the previous section, we argued that the small within-subject sample size and the relative lack of task-based fMRI are two major challenges in performing encoding model analysis on developmental CCN. Here we propose potential solutions to those issues.

First, the issue of small within-subject sample size can be overcome by concatenating data from different subjects, and encoding models can be trained based on the aggregated samples. When training such encoding models, cross-validation is performed in a cross-subject manner, unlike the single subject analysis where cross-validation is across trials or runs. Without any preprocessing, this may cause a reduction of prediction accuracy due to functional variability across subjects. To address this issue, researchers have developed the hyperalignment (functional alignment) technique (Haxby et al., 2020). Hyperalignment is a method that transforms the multivoxel activity patterns (within a target region-of-interest) of different individuals into a reference subject’s space by using Procrustes transformation (Goodall, 1991). The Procrustes transformation minimizes the distance between two given matrices (where rows correspond to stimuli or trials, and columns correspond to voxels or vertices) with a rigid transformation (consisting of rotation, reflection, scaling, and translation), enabling diverse brain activity patterns of multiple subjects to be aligned into a similar pattern. Using this technique, Haxby et al. (2011) reported a performance increase in between-subject object classification using fMRI data in the ventral temporal cortex compared to anatomical alignment. Guntupalli et al. (2016) extended this approach to searchlight spheres to enable a fine-grained transformation covering the whole cortex. Furthermore, Van Uden et al. (2018) trained encoding models on movie-viewing fMRI data and found that cross-subject prediction performance increased after the searchlight hyperalignment. Other recent studies have adopted different approaches to construct generalizable encoding models. For example, Nastase et al. (2020) transformed individuals’ response time series into shared feature space via functional connectivity and trained a semantic encoding model with the transformed time series. Gu et al. (2022) combined predicted responses of multiple subjects to further construct an ensemble encoding model predicting new subject’s data. These studies indicate the potential of incorporating multi-subject activity data to build more generalizable encoding models.

Second, the challenge of acquiring task-based neuroimaging data in pediatric populations could be overcome by the use of naturalistic paradigms. One promising analysis of naturalistic data is inter-subject correlation, which analyses how temporal activity patterns synchronize across different subjects. In inter-subject correlation analysis, correlations between time series of brain activity of different subjects evoked by the same stimulus (e.g., a movie) are calculated across all combinations of subject pairs (Nastase et al., 2019). For example, Amalric and Cantlon, (2023) used this method to analyze movie viewing task-fMRI data with children. Although the inter-subject correlation is a useful model-free method, it cannot decompose the original complex stimuli into cognitive components (visual, auditory, semantic, mathematics, etc.) and leaves it unclear how brain representations of these different components dynamically change during the experiment. Encoding models can address this issue by decomposing naturalistic stimuli into latent features with hundreds to thousands of dimensions and compare their prediction performance.

Furthermore, open data resources would accelerate developmental CCN research. In addition to limited types of task fMRI data published in Adolescent Brain Cognitive Development (ABCD) study (Casey et al., 2018) or in UK Biobank (Littlejohns et al., 2020), researchers have published a series of open task-fMRI datasets of school children in the domain of reading, arithmetic, and reasoning (Lytle et al., 2020, Lytle et al., 2019, Suárez-Pellicioni et al., 2019, Wang et al., 2022). Healthy Brain Network (Alexander et al., 2017) also provides naturalistic movie viewing task-fMRI data from a broad range of age groups (5- to 21-year-olds). The growth of public repositories for sharing data, such as OpenNeuro (Markiewicz et al., 2021), would provide opportunities for machine learning experts who do not have neuroimaging equipment but are interested in carrying out state-of-the-art analyses using developmental neuroimaging data. For example, two encoding model studies (Im et al., 2024, Kamps et al., 2022) were based on the publicly available dataset (https://openneuro.org/datasets/ds000228/versions/1.1.1). The availability of larger-scale data would lead to more accurate model building.

There is still room for improvement in the methodology of encoding models in developmental cognitive neuroscience. For example, when it comes to assessing individual growth over time, longitudinal data are the gold standard, as cross-sectional studies incur the risk of confounding development with cohort effects. However, a majority of developmental neuroimaging studies are cross-sectional (Battista et al., 2018, Fair et al., 2021), and developmental trajectory of longitudinal and cross-sectional brain data do not always converge (Keresztes et al., 2022, Nyberg et al., 2010). In this context, the study of Di Liberto et al. (2023) is instrumental as it shows how longitudinal data can be used to construct TRF models, so that developmental changes in prediction performance can be evaluated within individuals. Although in that study the authors have built their models independently at each age, it may be possible to go a step further and explicitly incorporate a developmental effect into the model (e.g., linear mixed effect models; Bernal-Rusiel et al., 2013). Such an approach would make it possible to estimate the impact of cohort effects on encoding models. The model training algorithm also has room for improvement. Although most previous encoding model studies used regularization algorithms, a recent study implemented a boosting algorithm that minimizes nonzero parameters (Brodbeck et al., 2023). These technological developments will help constructing increasingly accurate encoding models.

In the future, more widespread use of encoding models will facilitate comparisons between human brain development and artificial intelligence. This goes beyond mere human-machine comparisons to reveal how information on various cognitive functions is represented in the brain and how it dynamically changes with learning and development.

## 7 Conclusion

In conclusion, we discussed here the progress and challenges of the emerging field of developmental CCN, with a particular focus on encoding models. The developmental CCN will serve as a bridge between developmental science, cognitive science, neuroscience, and artificial intelligence, and will be the target of collaborative research between researchers from these different disciplines. Although previous studies suffer from small within-subject sample sizes and lack of task neuroimaging data, these challenges could be addressed by using functional alignment techniques and naturalistic paradigms. Such methodological advances would pave the way for computational modeling of neuro-cognitive development.

## CRediT authorship contribution statement

**Jérôme Prado:** Writing – review & editing, Supervision, Funding acquisition. **Charlotte Constant-Varlet:** Writing – review & editing. **Tomoya Nakai:** Writing – original draft, Visualization, Investigation, Conceptualization.

## Declaration of Competing Interest

The authors declare no competing interests.
